# Editorial: Microbial Exopolymers: Sources, Chemico-Physiological Properties, and Ecosystem Effects in the Marine Environment

**DOI:** 10.3389/fmicb.2018.01822

**Published:** 2018-08-08

**Authors:** Tony Gutierrez, Andreas Teske, Kai Ziervogel, Uta Passow, Antonietta Quigg

**Affiliations:** ^1^School of Engineering and Physical Sciences, Heriot-Watt University, Edinburgh, United Kingdom; ^2^UNC Marine Sciences, University of North Carolina at Chapel Hill, Chapel Hill, NC, United States; ^3^Institute for the Study of Earth, Oceans, and Space, University of New Hampshire, Durham, NH, United States; ^4^Marine Science Institute, University of California, Santa Barbara, Santa Barbara, CA, United States; ^5^Ocean Sciences, Memorial University of Newfoundland, St. John's, NL, Canada; ^6^Texas A&M University at Galveston, Galveston, TX, United States

**Keywords:** microbial exopolymers, phytoplankton, marine environment, marine snow, marine oil snow (MOS), Deepwater Horizon, exopolysaccharide (EPS)

A large proportion of the total carbon in the World Ocean is in the form of dissolved organic matter (DOM), which is comparable in mass to the carbon in atmospheric CO_2_ (Hansell and Carlson, 1998). A major source of this material derives from the synthesis and release of exopolymers, or extracellular polymeric substances (EPS), mainly by bacteria and eukaryotic phytoplankton (Verdugo, 1994; Aluwihare et al., 1997). An initial understanding on the secretion of EPS by microorganisms, and their potential stabilizing effects for microbial cells, emerged during the last century with the first report by ZoBell and Allen (1935). We now know that most bacteria, and other microorganisms, occur associated with biofilms, either attached to surfaces or as suspended-aggregates in the water column. Exopolymer secretions thus serve important functions in marine environments, where they may be involved in microbial adhesion to solid surfaces and biofilm formation (Thavasi and Banat, 2014). They have also been shown to be involved in emulsification of hydrocarbon oils to enhance biodegradation (Gutierrez et al., 2013), mediating the fate and mobility of heavy metals and trace metal nutrients (Bhaskar and Bhosle, 2005; Gutierrez et al., 2008, 2012), or interacting with dissolved and/or particulate organic matter (Long and Azam, 2001). This wide spectrum of functional activity is reflected not merely in the complex chemistry of these biopolymers, but also in the diversity of bacterial and phytoplankton genera found producing them.

In this special issue, nine articles highlight new findings on the sources, chemico-physiological properties, functions and ecosystem effects of microbial exopolymers, including their role in the Gulf of Mexico following the Deepwater Horizon oil spill disaster. To begin, the article by Decho and Gutierrez introduces this Research Topic with a comprehensive review on microbial-derived EPS, covering their wide chemical composition, their partitioning, turnover and roles within the marine water column and sediment, their provision of selective adaptations to the producing microorganisms and other organisms, including in animal-microbe interactions, and the broader influences of these diverse macromolecules upon ocean and planetary processes.

An important role of exopolymers is in forming marine snow—floating or suspended particles that harbor a community of microorganisms and drive the biological pump. They also play an essential role in marine oil snow (MOS) that forms as a consequence of oil spillage. The article by Sperling et al. reveals a correlation between the concentration of high-molecular-weight organic matter (HMW-OM) and the diversity of particle-associated bacteria during the development of a spring bloom in the southern North Sea, suggesting that the availability of carbohydrates contributes to the multifactorial control of marine bacterioplankton communities. The article by Suja et al. reports, for the first time, the use of Illumina MiSeq sequencing to analyse the bacterial communities associated with MOS. This study was conducted in subarctic waters of the northeast Atlantic, revealing the enrichment of oil-degrading and EPS-producing bacteria on MOS, and providing supporting evidence that MOS particles are “hotspots” of these organisms with potentially profound consequences to the oil biodegradation process in the water column. Since the seafloor is the final endpoint for MOS, the article by Yang et al. reports the distinct occurrence and patterns of oil-associated family- and genus-level bacterial groups over time in surficial sediments in the Gulf of Mexico following MOS sedimentation after the Deepwater Horizon oil spill, and suggest petrocarbon sedimentation and changing biogeochemical niches as factors that shape these bacterial communities in near-surface seafloor sediments.

A cluster of three articles (Nosaka et al.; Ortega-Retuerta et al.; Thornton et al.) focus on the origin, chemical composition, dynamics and distribution of transparent exopolymer particles (TEP), which are defined as >0.4-μm transparent particles and constitute an important component of EPS. TEP may promote marine snow formation, thereby influencing the efficiency of the biological pump. They serve as a labile source of carbon and energy to micro- and macro-heterotrophs, and are thus important in food webs. The article by Nosaka et al. (2017) focuses on TEP distribution and its potential correlation to diatom-derived chlorophyll-*a* biomass and DOC productivity during the spring bloom periods in 2010 and 2011 in surface waters of the Oyashio region of the western subarctic Pacific, where seasonal biological drawdown of the partial pressure of CO_2_ is one of the greatest in the world's oceans. The article by Ortega-Retuerta et al. describes TEP distributions in the northwest Mediterranean Sea during late spring 2012, along perpendicular and parallel transects to the Catalan coast, and its correlation with chlorophyll-*a*, O_2_ (as a proxy of primary production) and bacterial production. The paper by Thornton et al. focuses on the concentrations and potential correlation of TEP and Coomassie staining particles (CSP) in the sea surface microlayer and underlying water in the Pacific Ocean off the coast of Oregon (United States) during July 2011.

The production and utilization of fluorescent dissolved organic matter (DOM), and the influence of diatom-derived EPS in shaping microbial communities in marine waters are the research foci in the final two articles (Bohórquez et al.; Goto et al.), respectively. The paper by Goto et al. presents data linking bacterial growth and DOM production, of different recalcitrant characteristics, by the marine bacterial isolate *Alteromonas macleodii* as a model marine bacterium which is ubiquitously found from the sea surface to the deep waters of tropical and temperate oceans (López-Pérez et al., 2012). The article by Bohórquez et al. shows that fractions of EPS with different structural complexity (operationally termed colloidal and hot-bicarbonate extracted) in intertidal sediments are degraded at higher rates compared with those reported in previous studies, and contribute to the transfer of organic carbon from the microphytobenthos to heterotrophic bacteria.

In summary, these articles cover a range of roles and functions for microbial exopolymers in the marine environment. One challenge remains to develop methods that can accurately trace the production of exopolymers to their biological source in environmental samples. Such methods and approaches would be invaluable for understanding what type(s) of exopolymers participate in marine snow formation, as well as in the formation of MOS, and for consequently linking these exopolymers to their producing organism(s). We hope that this special issue stimulates more discussion and inspires new lines of research on the incredibly profound, and still largely enigmatic subject of microbial exopolymers.

## Author contributions

All authors listed have made a substantial, direct and intellectual contribution to the work, and approved it for publication.

### Conflict of interest statement

The authors declare that the research was conducted in the absence of any commercial or financial relationships that could be construed as a potential conflict of interest.
